# Sensory Characterization of Licorice Extract in Formulated Spirits and the Intervention of Puerarin on Sweetness Lingering

**DOI:** 10.3390/foods15132292

**Published:** 2026-06-26

**Authors:** Linfen Wu, Siqian Guo, Minxin Liu, Kexi Ma, Yu Lan, Jingming Li

**Affiliations:** 1 College of Food Science & Nutritional Engineering, China Agricultural University, No. 17 Tsinghua Dong Road, Haidian District, Beijing 100083, China; wulinfen0619@163.com (L.W.); guosiqian2022@163.com (S.G.); ancientmise@163.com (M.L.); 2 Sichuan Advanced Agricultural & Industrial Institute, China Agricultural University, Chengdu 611430, China; makexi2009@163.com; 3 Luzhou Laojiao Co., Ltd., Luzhou 646000, China; lanyu@lzlj.com

**Keywords:** glycyrrhizic acid, formulated spirits, sensory characteristics, puerarin, molecular docking

## Abstract

Glycyrrhizic acid (GA), a high-potency natural sweetener derived from licorice, has long been limited in alcoholic beverages due to its characteristic lingering sweetness in both aqueous and ethanol matrices. From an industrial perspective, licorice extract offers superior economic viability and processing efficiency compared to high-purity monomers. To clarify the sensory behaviour of licorice extract in formulated spirits, this study characterized the sensory attributes of licorice extract (containing 23.75% GA) in 42% and 52% vol base spirits. Quantitative results showed that the detection thresholds, recognition thresholds, and upper limits of comfort were 2.23, 15.45, and 75.13 mg/L in the 42% vol base spirit, and 5.28, 25.64, and 72.98 mg/L in the 52% vol base spirit, respectively. Suitable addition levels were identified as 30 mg/L for 42% vol and 40 mg/L for 52% vol base spirits. The relative sweetness of GA was determined to be 175.83 times that of sucrose. Sucrose showed a sweetness duration of 10 to 12 s, whereas licorice extract exceeded 16 s. Puerarin showed the strongest effect in mitigating lingering sweetness, reducing the sweetness duration to values comparable to those of sucrose at 50 mg/L in the water system and 30 mg/L in the 10% vol edible alcohol system. Molecular docking suggested that puerarin may interact more favourably with the sweet taste receptor than GA, with a binding energy of −52.72 kJ/mol, and may weaken the predicted GA–receptor interaction, as reflected by the shift in GA binding energy from −41.00 to −21.30 kJ/mol. Overall, this study provides sensory parameters specific to different ethanol matrices for applying licorice extract in formulated spirits and offers a plausible receptor-level explanation, supported by molecular docking, for the ability of puerarin to mitigate GA-induced lingering sweetness, thereby supporting the development of formulated spirits with reduced sugar content.

## 1. Introduction

Licorice is a traditional medicinal herb with various pharmacological activities, including anti-inflammatory, antiviral, immunomodulatory, and neuroprotective effects [1,2]. Since 2002, it has been officially listed as a dual-purpose medicinal and food ingredient and serves as the primary source of the natural high-potency sweetener glycyrrhizic acid (GA) [3]. GA possesses a sweetness intensity approximately 150–170 times that of sucrose [4,5], exhibits excellent solubility and thermal stability [6], and complies with the National Food Safety Standard for Food Additive Usage (GB 2760-2024) [7]. In practical applications, licorice extract offers superior economic viability compared to high-purity monomers due to its low-cost and efficient extraction processes [6,8,9]. Consequently, investigating the sensory characteristics of licorice extract in base spirits is not only of industrial significance but also serves as a practical foundation for elucidating the flavour patterns of GA.

Formulated spirits are alcoholic beverages produced by blending or further processing fermented alcoholic beverages, distilled spirits, or edible alcohol as the base, together with edible raw materials, auxiliary ingredients, or food additives [10]. In these products, the extensive use of animal- and plant-derived flavouring materials can introduce undesirable taste attributes, such as sourness, bitterness, and astringency, which are commonly balanced or masked by added sugars. Consequently, reducing sugar addition while maintaining flavour balance has become a major technical bottleneck for the formulated spirits industry. Owing to its high-potency sweetness, GA may attenuate off-taste notes as well as the pungency and drying sensation associated with high-alcohol base spirits through taste-masking effects. This enables improvement of mouthfeel smoothness and sensory balance in formulated spirits without substantially altering the original flavour structure of the base spirit [4,11]. Therefore, the use of GA in formulated spirits provides a technically feasible and industrially relevant approach for developing sugar-reduced alcoholic beverages.

However, the sweetness profile of licorice differs markedly from that of sucrose. Inappropriate addition levels often lead to undesirable sensations such as excessive sweetness and after-bitterness [12]. More importantly, the sweetness of GA is characterized by a delayed onset and prolonged persistence, often resulting in a cloying sensory experience [13]. This phenomenon, commonly referred to as lingering sweetness, is primarily attributed to the prolonged binding and retention of GA molecules on the taste buds. This problem restricts the broader application of licorice and its extracts in food and beverage systems, particularly in soft drinks and alcoholic beverages. At the molecular level, the prolonged sweetness perception of GA may be related to its strong interaction with sweet taste receptors and slow dissociation from the T1R2/T1R3 receptor complex [14]. Therefore, regulating the receptor-level interaction between GA and sweet taste receptors may provide a feasible strategy for mitigating lingering sweetness.

Sensory interactions offer an additional basis for this strategy. Sweetness can counteract bitterness, acidity, and astringency through antagonistic or masking effects, thereby contributing to a more balanced flavour profile [11]. Meanwhile, natural polyphenols with specific molecular structures may interact with taste receptors or interfere with the binding behaviour of high-potency sweeteners, suggesting their potential as modulators of sweetness persistence [15]. However, the sensory behaviour of licorice extract in ethanol matrices and the modulation of GA-related lingering sweetness by structurally distinct polyphenols at the receptor level remain insufficiently understood. Based on this research gap, the central research question of this study was whether natural polyphenols could mitigate the GA-related lingering sweetness and whether this effect could be interpreted at the receptor level. Therefore, the objective of this study was to characterize the sensory behaviour and applicable concentration range of licorice extract in formulated spirit bases and to evaluate the ability of selected natural polyphenols to reduce GA-related lingering sweetness. It was hypothesized that natural polyphenols with different molecular structures could mitigate GA-related lingering sweetness to varying extents, possibly by modulating the interaction between GA and sweet taste receptors. To test this hypothesis, this study first established sensory parameters of licorice extract across different ethanol matrices in formulated spirit bases, including sensory thresholds, comfort ranges, suitable addition levels, and sweetness duration. Catechin, EGCG, and puerarin were then compared as structurally distinct polyphenols, and their sensory effects were linked with receptor interactions predicted by molecular docking. Unlike conventional empirical flavour-masking approaches, this study connects sensory characterization with receptor interaction analysis, thereby providing practical formulation parameters for broadening the application of licorice extract in alcoholic beverages and developing sugar-reduced formulated spirits.

## 2. Materials and Methods

### 2.1. Materials and Reagents

Licorice (*Glycyrrhiza uralensis* Fisch.) was purchased from Gansu Minnongren Chinese Medicinal Materials Co., Ltd. (Dingxi, China). Chinese Baijiu base spirits (42% and 52% vol) were provided by Luzhou Laojiao Co., Ltd. (Luzhou, China). Food-grade sucrose was purchased from Nanjing Ganzhiyuan Sugar Co., Ltd. (Nanjing, China). Quinine hydrochloride (purity ≥ 99%) was obtained from J&K Scientific Ltd. (Beijing, China). Beidan No. 1 (commercial tannin) was sourced from Shanghai Dingtang International Trade Co., Ltd. (Shanghai, China). Macroporous resin (LX-60) was purchased from Shanghai Macklin Biochemical Technology Co., Ltd. (Shanghai, China). Analytical-grade potassium chloride (KCl) was purchased from Shanghai Yuanye Bio-Technology Co., Ltd. (Shanghai, China). Food-grade activated carbon was sourced from Pingdingshan Lvzhiyuan Activated Carbon Co., Ltd. (Pingdingshan, China). HPLC-grade ethanol, phosphoric acid, and acetonitrile were purchased from Shanghai ANPEL Laboratory Technologies Co., Ltd. (Shanghai, China). The GA standard (purity ≥ 98%) was obtained from Beijing BioDee Biotechnology Co., Ltd. (Beijing, China). Puerarin, catechin, and epigallocatechin gallate (EGCG) were purchased from Shanghai Aladdin Biochemical Technology Co., Ltd. (Shanghai, China). Edible alcohol (grain-based neutral spirit, ≥95% vol, complying with the national food safety standard GB/T 10343-2023 [16]) was obtained from Henan Xinheyang Alcohol Co., Ltd. (Jiaozuo, China). Purified drinking water used for sensory evaluation was purchased from Hangzhou Wahaha Group Co., Ltd. (Hangzhou, China). Ultrapure water used for HPLC and electronic tongue analyses was prepared using a laboratory ultrapure water purification system.

### 2.2. Methods

#### 2.2.1. Preparation and Purification of Licorice Extract

The preparation and purification of licorice extract were conducted according to the modified methods of Wan et al. [8,17]. Specifically, the crude licorice extract was obtained via thermal maceration in an NG-5000 digital thermostatic water bath (Sartorius AG, Göttingen, Germany) with a solid-to-liquid ratio of 35:1 at 55 °C for 30 min. The resulting extract was then purified using LX-60 macroporous resin (Shanghai Macklin Biochemical Technology Co., Ltd., Shanghai, China) with a total bed volume (BV, defined as the volume occupied by the packed resin) of 20 mL. Both the loading and elution flow rates were maintained at 2 BV/h (equivalent to 40 mL/h) using a BT100-2J/DG-2 peristaltic pump (Longer Precision Pump Co., Ltd., Baoding, China), with a loading volume of 20 BV (totaling 400 mL). A 50% ethanol solution was employed as the eluent, and this purification procedure was repeated once to ensure high purity. Subsequently, the purified eluate was decolorized by adding 2% activated carbon at 40 °C for 40 min. Following filtration to remove the activated carbon, the filtrate was concentrated under reduced pressure using a rotary evaporator connected to an I-100 circulating water multi-purpose vacuum pump (Büchi Labortechnik AG, Flawil, Switzerland). The resulting concentrate was subsequently dried in a DHG-9140A electric thermostatic blast drying oven (Beijing Boyu Baowei Experimental Equipment Co., Ltd., Beijing, China) to obtain the final licorice extract.

#### 2.2.2. High-Performance Liquid Chromatography (HPLC) Analysis of GA

The analysis of GA was performed according to the method described by Sun et al. [18]. Chromatographic separation and detection were conducted using an Agilent 1100 high-performance liquid chromatography (HPLC) system equipped with a diode-array detector (DAD) and a ZORBAX SB-C18 column (4.6 mm × 250 mm, 5 µm), all from Agilent Technologies (Santa Clara, CA, USA). The detection wavelength was set at 250 nm. The mobile phase consisted of 0.1% phosphoric acid (A) and acetonitrile (B). The gradient elution program was executed as follows: 0–3 min, 62–50% A; 3–10 min, 50–48% A; 10–11 min, 48–62% A; 11–16 min, 62% A.

Qualitative Analysis: Identification was achieved by comparing the retention times of the licorice extract peaks with those of the GA standard in the resulting chromatograms.

Quantitative Determination: The licorice extract was dissolved in 70% ethanol-water solution to prepare a test solution with an extract concentration of 40.0 mg/L. After filtration through a 0.22 μm organic microporous membrane, the test solution was subjected to HPLC analysis. The GA content in licorice extract was quantified using an external standard method based on an HPLC calibration curve established with GA standard solutions. Methodological validation was performed for the GA determination method [19]. The calibration curve was y = 25,128x − 121,969, with a coefficient of determination of *R*^2^ = 0.9982, indicating good linearity within the tested concentration range. The limit of detection (LOD) and limit of quantification (LOQ) were 0.23 mg/L and 1.29 mg/L, respectively. The precision of the method was evaluated using intra-day and inter-day relative standard deviations (RSDs), which were 1.55% and 1.49%, respectively. Accuracy was assessed by recovery tests at three spiking levels, namely 50%, 100%, and 200%, yielding an average recovery of 101.5%, with values ranging from 100.8% to 102.4%. These validation results confirmed that the method showed acceptable linearity, sensitivity, precision, and accuracy for GA quantification in licorice extract. The GA content was calculated according to the calibration curve, and the determination was performed in triplicate (n = 3). The purity of GA was calculated using Equation (1), and the final result was expressed as the mean ± standard deviation (SD).

(1) $$F=\frac{C_{GA}V_{1}V_{3}}{mV_{2}}\times 100\%$$

where *F* is the purity of GA in the licorice extract (%), *C*_GA_ is the mass concentration of GA (mg/L), *V*_1_ is the constant volume of the sample stock solution (L), *V*_2_ is the volume of the stock solution aliquot accurately pipetted (mL), *V*_3_ is the final constant volume after the second dilution (mL), and *m* is the initial mass of the licorice extract (mg).

#### 2.2.3. Selection and Training of Sensory Panelists

Volunteers aged 18 to 45 were recruited from the College of Food Science and Nutritional Engineering at China Agricultural University via electronic questionnaires. During the initial screening, the volunteers’ taste identification ability and sensory sensitivity were evaluated in accordance with GB/T 16291.1-2012, titled Sensory analysis: General guidance for the selection, training and monitoring of assessors, Part 1: Selected assessors [20]. A minimum accuracy rate of 80% was required for volunteers to be considered qualified.

The qualified candidates underwent a cumulative 30 h intensive training program in accordance with GB/T 15549-2022 [21] and GB/T 12312-2012 [22]. To ensure reproducibility across different matrices, a “water-to-spirit” adaptation protocol was implemented, training panelists to recognize sweetness in progressively complex systems ranging from aqueous solutions to 52% vol alcohol bases. Under this protocol, the training curriculum encompassed different testing, olfactory and gustatory sensitivity measurements, sensory attribute identification, and categorical scaling methodologies. The validation was benchmarked using a standardized sensory series (referred to as G3-level intensity in the internal protocol), defined as 8.0 g/L sucrose (sweetness), 0.005 g/L quinine hydrochloride (bitterness), and 0.20 g/L tannins (Beidan No. 1, astringency) in a spirit-simulated matrix. Following the training period, a comprehensive assessment was conducted based on GB/T 10220-2012 [23]. Panelist consistency was rigorously validated through ranking tests for sweetness intensity, where intra-individual reproducibility required a correlation coefficient r > 0.85 across three independent sessions, and inter-individual consensus was ensured by excluding candidates whose scaling scores for primary attributes exceeded the triple standard deviation (±3σ) of the group mean.

A total of 53 individuals successfully met these stringent validation benchmarks to constitute the final sensory panel, which comprised 28 females and 25 males. The study was conducted in accordance with the Declaration of Helsinki and approved by the Ethics Committee for Human Research of China Agricultural University (protocol code CAUHR-20240904, date of approval: 1 September 2024). All participants provided informed consent and signed written consent forms prior to their involvement in the study.

#### 2.2.4. Sensory Evaluation Methodology for Determining Suitable Addition Levels

Sensory descriptors were screened and established by the panel in accordance with GB/T 33405-2016 [24] and GB/T 33404-2016 [25]. Sweetness, mouthfeel, bitterness, and off-flavour were selected as the primary attributes for evaluation. Sweetness was selected to assess the sweetening contribution of licorice extract, while mouthfeel was used to evaluate its potential effect on masking the pungency, burning sensation, and drying sensation associated with high-alcohol base spirits. Bitterness and off-flavour were considered because these undesirable notes may be introduced when licorice extract is added at inappropriate levels. A 0–5 categorical scoring scale was employed for all sensory attributes according to predefined criteria [26]. For mouthfeel, higher scores indicated a smoother and less irritating oral sensation, whereas lower scores indicated greater mouthfeel irritation. For the remaining attributes, scores increased with perceived intensity. This scoring scale was selected to compare sensory differences among formulations and to identify suitable addition levels of licorice extract. Because high-alcohol matrices such as 42% and 52% vol Baijiu elicit pronounced trigeminal stimulation, including burning and stinging sensations [27], the categorical scoring scale was adopted to reduce sensory fatigue and maintain panelist reproducibility under high-alcohol tasting conditions. The relatively large number of validated assessors was used to improve the reliability of the categorical sensory scores. A double-blind experimental design was implemented using specialized spirit tasting glasses. Each sample, consisting of 15 mL of liquid, was labeled with a 3-digit random code and presented to the panelists in a balanced order. All panelists were required to abstain from pungent or strongly flavoured foods for at least 2 h prior to the session. During sensory evaluation, panelists were instructed to take the sample into the mouth and distribute it evenly throughout the oral cavity by gentle tongue movement, allowing the liquid to cover the surface and posterior region of the tongue and to fully contact the oral mucosa. The sample was held in the mouth for 3 to 5 s to allow perception of taste and mouthfeel attributes, and was then expectorated. After expectoration, panelists gently inhaled through the mouth and exhaled through the nose to facilitate retronasal perception of volatiles and aftertaste assessment. The final attribute scores were assigned based on the integrated perception of aroma, taste, and mouthfeel. After each sample evaluation, panelists rinsed their mouths thoroughly and rested for 5 min until the palate returned to a neutral state before evaluating the next sample. Each continuous evaluation session was limited to 30 min. All samples were evaluated in triplicate, and the mean values were used as the final scores.

#### 2.2.5. Determination of the Relative Sweetness of Licorice Extract

##### 2-Alternative Forced Choice (2-AFC) Method

The sweetness intensity of the licorice extract was determined using the 2-AFC method according to the procedures described by Kim et al. [28,29]. Aqueous solutions of the licorice extract were prepared at various concentrations, while a 10 g/L sucrose solution served as the reference. The 53 validated members of the sensory panel compared the sweetness intensity of each sample against the control. Based on the sensory evaluation data, concentration-response (C-R) curves were constructed to model the relationship between extract concentration and perceived sweetness. The 2-AFC method was selected over the 3-AFC or triangle tests because it provides higher statistical power for directional intensity comparisons between a specific sweetener and a reference [30]. To model the C-R relationship, a quadratic regression model was employed. This model was chosen because high-potency sweeteners often exhibit non-linear intensity growth at higher concentrations, and it provides a more accurate estimation of the equi-sweetness concentration (the point where the sample and the 10 g/L sucrose reference are perceived as equally sweet) compared to simple linear models. The relative sweetness of the licorice extract was subsequently derived using Equation (2), and the relative sweetness of GA was calculated according to Equation (3).

(2) $${RS}_{LE}=\frac{C_{sugar}}{X}$$

where *RS*_LE_ is the relative sweetness of the licorice extract, *C*_sugar_ is the concentration of the reference sucrose solution (g/L), and *X* is the calculated concentration of the extract corresponding to a 50% response rate (g/L).

(3) $${RS}_{GA}=\frac{{RS}_{LE}}{F}$$

where *RS*_GA_ is the relative sweetness of GA, *RS*_LE_ is the relative sweetness of the licorice extract, and *F* is the purity of GA within the extract.

##### Electronic Tongue Analysis for Relative Sweetness Determination

Sweetness evaluation was assisted by a TS-5000Z electronic tongue system from Intelligent Sensor Technology, Inc. (INSENT, Atsugi, Japan). Specific concentrations of licorice extract and sucrose solutions were prepared using a 10 mM potassium chloride (KCl) solution. Before the commencement of the experiment, the sensors underwent a rigorous cleaning protocol consisting of a 90 s rinse in positive cleaning solutions, followed by a 120 s stabilization in a reference solution. This cleaning procedure was then repeated in an additional reference solution. An Ag/AgCl reference electrode was utilized, and the measurement sequence was initiated only after the sensors achieved equilibrium in the reference solution. Each sample was measured four consecutive times, and the sensor response intensity at 30 s was recorded for each measurement. The results from the final three measurements were averaged as the representative value for each sample. The experimental data were processed via the INSENT taste analysis system to compare the sweetness scores of the sucrose reference and the licorice extract, which facilitated the determination of the relative sweetness of the extract.

#### 2.2.6. Determination of Thresholds and Comfort Ranges of Licorice Extract in Base Spirits

The detection thresholds, recognition thresholds, and comfort ranges of licorice extract in two base spirits with different ethanol concentrations (42% and 52% vol) were determined according to the modified methodologies of Nagai and Ebbeling [31,32]. Stock solutions of licorice extract (0.4 g/L) were prepared using the respective base spirits as solvents and were subsequently diluted to obtain a series of test samples. These samples were labeled with three-digit random codes, and panelists recorded their sensory responses accordingly. The detection threshold was calculated as the weighted geometric mean between the highest concentration detected by fewer than 50% of the panelists and the lowest concentration detected by more than 50% of the panelists, as shown in Equation (4). The recognition threshold, defined as the concentration at which the sweetness of licorice extract could be clearly identified, was determined using the same principle [5]. The comfort range was defined as the concentration interval between the recognition threshold and the upper limit of comfort. The upper limit of comfort was defined as the transition concentration beyond which sensory discomfort, such as cloying lingering sweetness or off-flavours, began to occur. It was calculated as the weighted geometric mean between the highest concentration at which fewer than half of the panelists reported discomfort and the lowest concentration at which more than half reported discomfort. Therefore, the comfort range represents a sensory-acceptable concentration interval in which licorice extract provides identifiable sweetness without exceeding the sensory tolerance limit [33].

(4) $$G=\sqrt[{\left(f_{1}+{\;f}_{2}\right)}]{x_{1}^{f_{1}}\times x_{2}^{f_{2}}}$$

where *G* represents the geometric mean, corresponding to the detection threshold, recognition threshold, or comfort limit; *f*_1_ and *f*_2_ denote the number of panelists who perceived, recognized, or reported discomfort; and *x*_1_ and *x*_2_ are the corresponding concentrations associated with these sensory responses.

#### 2.2.7. Duration of Sweetness Perception of Licorice Extract in Different Base Spirits

The sweetness persistence of licorice extract across varying ethanol concentrations was evaluated by recording the duration of perceived sweetness. A reference solution containing 2.4 g/L sucrose in base spirits and a gradient series of licorice extract solutions (at concentrations of 20, 30, 40, and 50 mg/L) were prepared. These samples were presented to the panelists in a randomized order. Panelists were instructed to introduce the sample into the oral cavity and facilitate comprehensive contact with the oral mucosa via tongue agitation for 3 to 5 s prior to expectoration. The duration was timed from the initial onset of perceived sweetness until its complete disappearance. To ensure sensory recovery and prevent carry-over effects, panelists rinsed their mouths with purified water and rested for 5 min between sample evaluations. Upon completion of the sessions, outliers were identified and excluded using the triple standard deviation method. The arithmetic mean was subsequently calculated to determine the final sweetness duration for each sample.

#### 2.2.8. Validation and Mitigation of Lingering Sweetness of Licorice Extract

To eliminate potential interference from the complex components present in base spirits and further validate the lingering sweetness of the licorice extract in simplified aqueous and ethanol matrices, sucrose solutions of equivalent sweetness were utilized as references. Based on the relative sweetness values determined in Section 2.2.5, sucrose equivalent conversions were performed to prepare aqueous solutions containing 192 mg/L licorice extract (equivalent to 8 g/L sucrose) and ethanol or base spirit solutions containing 48 mg/L licorice extract (equivalent to 2 g/L sucrose). The ethanol solutions, such as the 10% vol system used for screening, were prepared by diluting the edible alcohol with purified water. Specific quantities of natural polyphenols were subsequently incorporated into selected samples to evaluate their potential for sensory improvement. Targeted sensory attribute assessment was conducted to quantify the intensities of sweetness and bitterness. The duration of sweetness perception for each sample was recorded, and outliers were excluded according to the triple standard deviation method. The mean values were then calculated to provide the final results for each experimental group.

#### 2.2.9. Homology Modeling and Molecular Docking of Human Sweet Taste Receptors

The amino acid sequences of the human sweet taste receptors T1R2 and T1R3 were obtained from the NCBI database. Following a BLASTp search using NCBI BLAST+ v2.15.0+ against the PDB database, homologous proteins with high similarity were identified and screened as potential templates. Further template selection and homology modeling were conducted using Modeller 10.4 software and the SWISS-MODEL web server (https://swissmodel.expasy.org/). The initially constructed models were subsequently imported into SYBYL-X 2.1.1 software for energy minimization. The structural integrity and stereochemical quality of the resulting models were evaluated via SAVES v6.0. Models demonstrating high Verify 3D scores and reasonable conformations in Ramachandran plots were selected for further molecular docking investigations.

The 3D structures of GA and the selected polyphenols were constructed using Chem3D 19.0 software and subjected to energy minimization to achieve stable conformations. The optimized small molecules were then imported into AutoDockTools 1.5.6 for ligand preparation. Simultaneously, the structures of the receptor proteins were preprocessed using PyMOL 2.3.0 and AutoDockTools 1.5.6 to ensure proper atom typing and charge assignment. Potential binding sites within the protein structures were predicted via POCASA 1.1. Subsequently, molecular docking simulations were executed using AutoDock Vina 1.1.2 to explore the binding affinities and modes. The grid box was centered on the predicted binding site within the Transmembrane Domain (TMD) of the T1R2 and T1R3 subunits, using a default grid spacing of 1.0 Å to ensure comprehensive coverage of the helical cavities. To ensure the reproducibility and accuracy of the global minimum search, the exhaustiveness parameter was set to 20. The docking plausibility was assessed by checking whether the resulting binding configurations were consistent with established interaction patterns of G protein-coupled receptors (GPCRs) and maintained reasonable conformations. The resulting docking configurations and intermolecular interactions were visualized and analyzed using PyMOL and LigPlot^+^ v.2.2.

#### 2.2.10. Statistical Analysis

All experimental measurements and sensory evaluations were performed in triplicate, with analytical data expressed as the mean ± standard deviation (SD), and method reproducibility was evaluated using the relative standard deviation (RSD). Sensory thresholds and comfort limits were calculated from the percentages of panelists who detected, recognized, or reported discomfort at adjacent concentrations using weighted geometric means, as described in Section 2.2.6. Outliers in sweetness duration measurements were excluded using the triple standard deviation method. The concentration-response (C-R) curves for relative sweetness determination were fitted using a quadratic regression model, and the goodness-of-fit was evaluated by the coefficient of determination (*R*^2^). The coefficient of variation (CV) was calculated to describe inter-individual variability in sweetness duration. Descriptive statistics and quadratic regression analysis were performed using SPSS 20.0 software (SPSS Inc., Chicago, IL, USA). Graphical plotting was performed using Origin 2021 software (OriginLab Corporation, Northampton, MA, USA).

## 3. Results and Discussion

### 3.1. Determination of GA Purity in Licorice Extract

**Figure 1:**
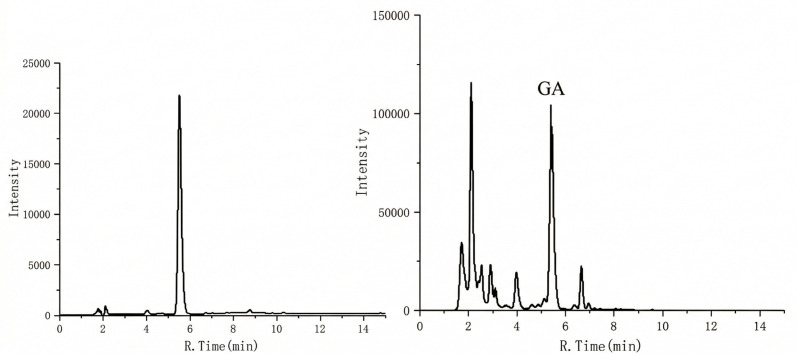
High-performance liquid chromatography (HPLC) chromatograms of glycyrrhizic acid (GA) standard and licorice extract.

The chromatograms of the GA standard and the licorice extract are presented in Figure 1. The target peak in the licorice extract was identified as GA by comparing its chromatographic retention time with that of the GA standard. The chromatographic profile showed that the GA peak was clearly distinguishable from adjacent components, supporting the subsequent quantitative determination. Based on the validated HPLC external standard method, GA purity in the licorice extract was calculated using Equation (1) and determined to be 23.75 ± 0.41%.

### 3.2. Relative Sweetness Intensity of Licorice Extract

The sweetness intensity of the licorice extract was determined using both sensory evaluation and electronic tongue analysis. Regression analysis was performed on the sensory results based on the 2-AFC method to establish a C-R curve. The resulting quadratic equation was y = 28.007x^2^ − 6.1607x + 0.3695 (*R*^2^ = 0.9913). The high R^2^ value indicated an excellent fit of the quadratic model to the aggregate sensory response for the relative sweetness of licorice extract. While individual sweet taste thresholds vary biologically, the panel’s aggregate response followed a clear, predictable relationship, suggesting that the intensive training effectively minimized random perceptual errors. The quadratic model demonstrated a superior fit compared to the simple linear model (*R*^2^ = 0.8443) and the traditional Weber-Fechner logarithmic model (*R*^2^ = 0.7037). This indicates that the perceived sweetness intensity of licorice extract increases more rapidly at higher concentrations, following a trend consistent with Stevens’ Power Law for high-potency sweeteners. The robustness of this regression supports that the subsequent derivation of relative sweetness is highly representative of the panel’s aggregate perception. Based on the equation, the concentration of the licorice extract at a 50% response rate was calculated to be 239.44 mg/L, representing a sweetness intensity equivalent to that of a 10 g/L sucrose solution. According to Equation (2), the sweetness of the licorice extract was 41.76 times that of sucrose. By adjusting for purity, the relative sweetness of GA was determined to be approximately 175.83 times that of sucrose, which is slightly higher than the reported range of 150 to 170 found in existing literature [4,5]. This discrepancy may be attributed to other sweetening components within the extract contributing to the overall perceived sweetness [5]. Finally, the electronic tongue results showed a relative sweetness of 39.52, generally supporting the sensory evaluation result of 41.76. The slight discrepancy between the electronic tongue and human sensory evaluation warrants a critical analysis of their respective sensing mechanisms. The electronic tongue relies on lipid/polymer membrane sensors that measure electrochemical potential changes on the surface, which is a purely physicochemical response [34]. In contrast, human sweetness perception is a biological process mediated by T1R2/T1R3 GPCRs, involving complex neural signaling and psychophysical factors [35]. Furthermore, the licorice extract contains trace saponins and synergists that might exhibit higher binding affinities for biological receptors than for the synthetic membranes of the e-tongue. The higher values observed in sensory evaluation suggest that the synergistic effects of multicomponent extracts are more effectively captured by the human olfactory-gustatory system than by current sensor technology [36,37].

### 3.3. Detection Thresholds and Comfort Ranges of Licorice Extract in Different Base Spirits

**Figure 2:**
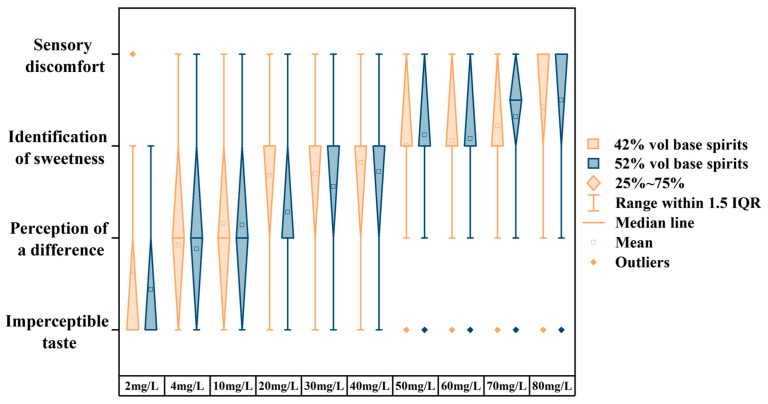
Sensory thresholds and comfort ranges of licorice extract. Orange and blue boxes denote the 42% vol and 52% vol base spirits, respectively. Except for these two group identifiers, the remaining legend items are general boxplot elements and are not color-specific. IQR, interquartile range.

As a natural high-potency sweetener, the excessive addition of the extract in alcohol and aqueous matrices can result in a prolonged lingering sweetness perception. Consequently, the sensory thresholds and upper comfort limits for the addition of the licorice extract were determined in this study, with the results illustrated in Figure 2. In the 42% vol base spirit, 44% of the panelists were able to perceive a difference at a licorice extract concentration of 2 mg/L. When the concentration reached 4 mg/L or higher, 62% of the panelists could perceive a difference or identify sweetness. Conversely, in the 52% vol base spirit, 46% of the panelists were unable to perceive a difference at 4 mg/L. At licorice extract concentrations of 10 mg/L and 20 mg/L, 38% and 64% of the panelists in the 42% vol base spirit perceived sweetness, respectively, while 70% and 38% of the panelists in the 52% vol base spirit were able to detect a difference or perceive sweetness. Based on Equation (4), the detection thresholds, recognition thresholds, and upper limits of comfort for the licorice extract in the 42% vol and 52% vol base spirits were 2.23, 15.45, and 75.13 mg/L, and 5.28, 25.64, and 72.98 mg/L, respectively. Compared with the 52% vol base spirit, the 42% vol base spirit showed lower detection and recognition thresholds for licorice extract, together with a slightly higher upper limit of comfort. This result suggests that the lower-ethanol matrix may reduce sensory masking and facilitate sweetness perception, thereby broadening the acceptable concentration range for formulation compared with higher-alcohol environments. Mechanistically, ethanol exerts a multifaceted influence on the perception of flavour substances. It has been reported that ethanol directly stimulates the sweet taste centers of the gustatory nerves, which can enhance the perception of sweeteners at lower concentrations. Furthermore, some studies suggest that sweetness and ethanol act on the same T1R2/T1R3 receptor, leading to a taste additive effect in lower alcohol systems [38]. Conversely, at higher ethanol concentrations (such as 52% vol), the intrinsic bitterness and trigeminal sensations (e.g., burning and stinging) associated with alcohol can exert a masking effect on sweet taste receptors [39], thereby elevating the detection and recognition thresholds of the licorice extract. Additionally, changes in ethanol concentration alter the solvent’s polarity and interaction kinetics, potentially affecting the binding stability of GA molecules within the TMD cavity of the sweet taste receptors [40]. Accordingly, the comfort ranges obtained in the two base spirits provide ethanol-matrix-specific formulation references for selecting suitable addition levels of licorice extract in formulated spirits. These ranges help balance identifiable sweetness and mouthfeel modulation with sensory acceptability, while reducing the risk of excessive lingering sweetness or off-flavours caused by over-addition.

### 3.4. Effects of Licorice Extract on Targeted Sensory Attributes of Base Spirits

**Figure 3:**
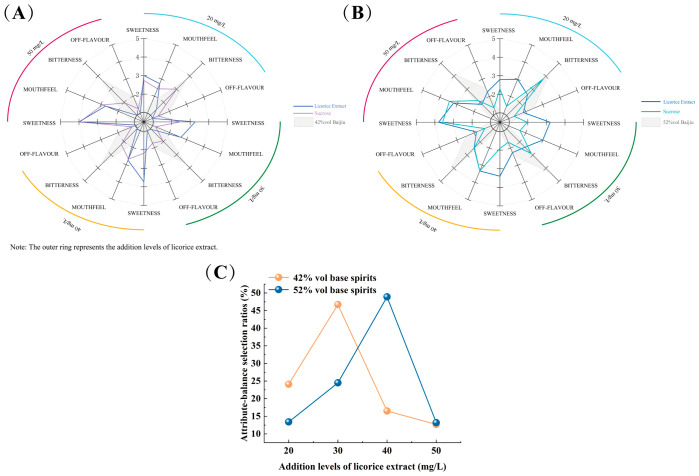
Effects of licorice extract on the targeted sensory attribute profiles of (**A**) 42% vol and (**B**) 52% vol base spirits and (**C**) attribute-balance selection ratios at different addition levels.

Using base spirits without sweeteners and samples with equivalent sucrose sweetness as controls, targeted sensory attribute assessment was performed to evaluate sweetness, mouthfeel, bitterness, and off-flavour in samples containing different concentrations of licorice extract. The resulting sensory attribute profiles are illustrated in Figure 3A,B. The addition of both sucrose and licorice extract increased the perceived sweetness of the base spirits while reducing the perceived bitterness. When comparing the scores of sucrose and licorice extract at equivalent sweetness levels within the same base spirits, the licorice extract exhibited a more pronounced masking effect on bitterness. Furthermore, the off-flavours and the sensory differences between the extract and sucrose were more pronounced in the higher alcohol spirit. Comparing identical concentrations of licorice extract across the two ethanol concentrations revealed that the intensities of bitterness and off-flavours in the 42% vol base spirit were lower than those in the 52% vol spirit. This finding suggested that higher alcohol concentrations may exert a masking effect on sweetness perception. In addition to the bitterness inherent to high alcohol content, the polyphenols present as impurities in the licorice extract may also serve as primary contributors to the perceived bitterness. The intensities of bitterness and off-flavours were notably more prominent in the 52% vol matrix. Figure 3C presents the attribute-balance selection ratios for different concentrations of licorice extract in the two base spirits. The sensory-balance criterion was mainly defined by a smoother mouthfeel, moderate sweetness, and lower bitterness and off-flavour intensities. Based on this criterion, 30 mg/L and 40 mg/L were selected as suitable addition levels for the 42% vol and 52% vol base spirits, respectively.

### 3.5. Sweetness Duration and Validation of Lingering Sweetness of Licorice Extract in Different Base Spirits

**Figure 4:**
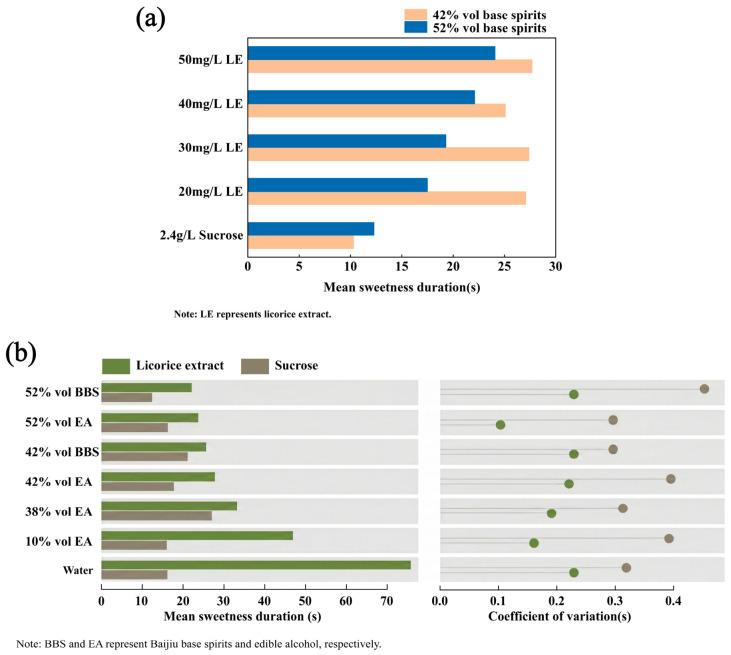
(**a**) Sweetness duration of licorice extract in base spirits and (**b**) the impact of matrix on sweetness duration. LE, licorice extract; BBS, Baijiu base spirits; EA, edible alcohol.

The results for the sweetness duration of the licorice extract across different ethanol concentrations are presented in Figure 4a. In both base spirits, the sweetness duration of sucrose was approximately 10 to 12 s, whereas the duration for the licorice extract exceeded 16 s. At the same ethanol concentration, the sweetness duration increased as the concentration of the licorice extract rose. Furthermore, the sweetness duration of the extract was notably longer in the lower alcohol concentration environment. Previous research has also demonstrated that high-potency sweeteners, whether synthetic or natural, generally exhibit a lower sweetness peak and a slow-onset, lingering aftertaste. This phenomenon is attributed to the high affinity of non-nutritive sweeteners for the binding sites of taste receptors, which results in a prolonged perception of lingering sweetness [41,42].

In addition to assessing the sensory characteristics of the licorice extract in base spirits, this study analyzed its lingering sweetness effect in a simplified edible alcohol system. Samples of base spirits and edible alcohol containing equivalent amounts of the extract and sucrose were evaluated to record the sweetness duration and calculate the coefficient of variation (CV), with results presented in Figure 4b. It was observed that the sweetness duration of the licorice extract was notably longer than that of sucrose in both the Baijiu base spirits and edible alcohol systems at identical alcohol concentrations. This indicated that the lingering sweetness perception of the licorice extract persisted across both complex spirit matrices and simplified alcohol systems. As the ethanol concentration increased, the lingering effect decreased, which was consistent with the previous findings that the lingering phenomenon was more pronounced in lower-alcohol bases. Compared to the alcohol-based systems, the lingering effect of the licorice extract was more evident in water. The CV for the sweetness duration of each sample was calculated to assess inter-individual variability. The CV values mostly fell within the range of 0.1 to 0.4, which represents a relatively narrow distribution for a large panel (n = 53) evaluating temporal sensory attributes. This suggests that the intensive training minimized the impact of individual biological differences in sweetness sensitivity. Notably, the CV was lower at lower alcohol concentrations, indicating that high-ethanol matrices may introduce additional sensory noise, whereas simpler matrices allowed for higher panelist consistency. Furthermore, the relatively lower CV values indicated a more consistent perception of licorice extract sweetness duration compared with sucrose. Overall, the relatively stable CV values across samples supported the reliability and consensus of the sensory panel in quantifying the lingering sweetness effect. To facilitate subsequent sensory evaluations and minimize interference from high alcohol content while establishing a more sensitive screening model, the water and 10% vol edible alcohol systems were selected to investigate the intervention of the lingering sweetness effect of the licorice extract.

### 3.6. Intervention of Polyphenols with Different Structural Characteristics on Lingering Sweetness Perception

Polyphenols are widely distributed in beverages such as fruit juices and wines, and contribute to sensory attributes and bioactivities such as antioxidant, anti-inflammatory, and vascular-protective effects [43]. To systematically investigate the intervention patterns of polyphenols on the lingering sweetness of the licorice extract, three representative polyphenols, namely catechin, EGCG, and puerarin, were strategically selected based on their distinct chemical skeletons, molecular complexities, and hypothesized inhibitory mechanisms. Specifically, catechin (a simple flavanol monomer) was selected to verify the sensory antagonistic effects associated with its significant sweet aftertaste characteristics [44,45]. EGCG, as an esterified flavanol with a galloyl group, was chosen due to its larger molecular size and complex structure to investigate the impact of greater steric hindrance on taste signaling [46]. Puerarin, a characteristic isoflavone with a unique C-glucoside skeleton, was employed to explore the specific regulatory capacity of its rigid structure on the TMD region of the sweet taste receptors [47]. By comparing the intervention effects of varying molecular complexities (monomers and esterified complexes) and chemical skeletons (flavanols and isoflavones), this model provides a useful framework for exploring how polyphenols may modulate sweetness perception at the molecular level.

**Figure 5:**
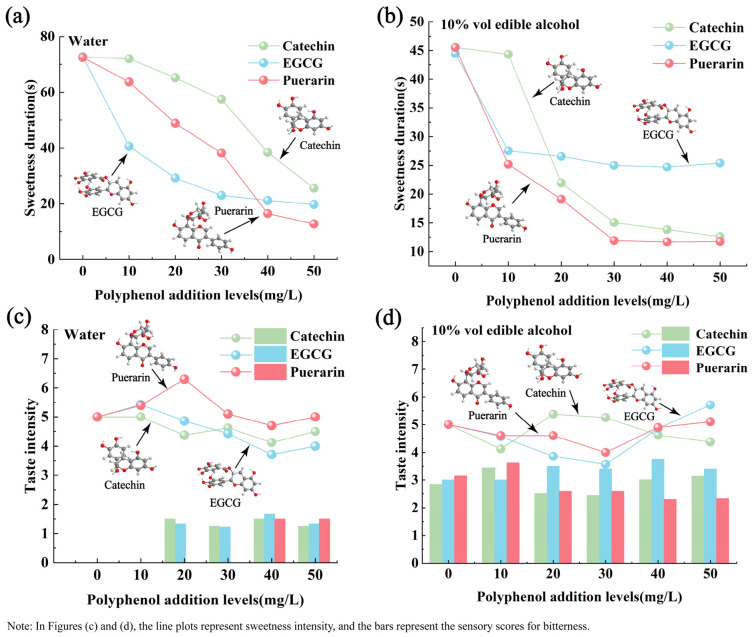
Effects of botanical polyphenols on the sweetness duration and taste intensity of licorice extract in different matrices: sweetness duration in the water system (**a**) and the 10% vol edible alcohol system (**b**); taste intensity in the water system (**c**) and the 10% vol edible alcohol system (**d**). EGCG, epigallocatechin gallate.

Polyphenols were added to the water and 10% vol edible alcohol systems containing a constant concentration of the licorice extract. The sensory panel evaluated the sweetness duration (Figure 5a,b) and taste intensity (Figure 5c,d). The addition of botanical polyphenols markedly reduced the sweetness duration of the licorice extract in both systems, with puerarin showing the strongest overall inhibitory effect. As shown in Figure 5a,b, in the water system, the sweetness duration of the sample was essentially identical to that of sucrose when puerarin was added at 50 mg/L. In contrast, only 30 mg/L puerarin was required to achieve the same effect in the 10% vol edible alcohol system. While reducing the sweetness duration, the incorporation of polyphenols also introduced bitterness. As shown in Figure 5c, sensory scoring revealed that polyphenols had only a limited impact on sweetness intensity in the water system. However, bitterness became perceptible when catechin and EGCG were added at concentrations exceeding 15 mg/L and when the puerarin addition level exceeded 40 mg/L, although the intensity remained relatively low. As shown in Figure 5d, changes in taste intensity were more pronounced in the 10% vol edible alcohol system. The effect of EGCG on lingering sweetness was limited, as it only reduced the duration to approximately 27 s while simultaneously decreasing sweetness intensity and introducing noticeable bitterness. By comparison, catechin and puerarin showed stronger effects in mitigating lingering sweetness in this system. At an addition level of 30 mg/L, both compounds effectively reduced the sweetness duration to a level comparable to that of sucrose.

These results were obtained in simplified water and 10% vol edible alcohol systems to reduce background interference and compare the effects of different polyphenols more clearly. In commercial formulated spirits, however, volatile and non-volatile constituents, such as aroma compounds, organic acids, phenolics, sugars, and other flavouring materials, may further influence sweetness perception through aroma–taste interactions, trigeminal stimulation, bitterness or astringency, and changes in matrix polarity [48]. Therefore, although puerarin showed the best overall performance in the simplified systems, the practical effectiveness of these polyphenols in reducing GA-related lingering sweetness in real formulated spirit matrices should be further validated under product-level formulation and storage conditions.

### 3.7. Homology Modeling of Human Sweet Taste Receptors

**Figure 6:**
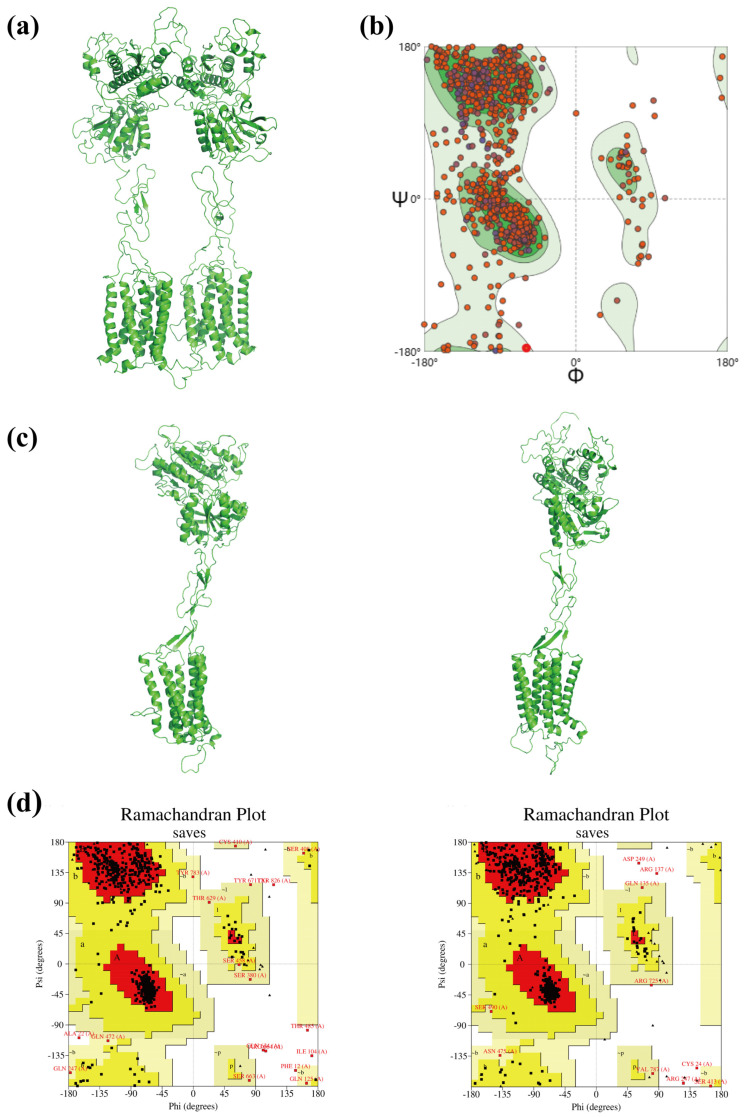
Homology modeling and structural validation of sweet taste receptors T1R2/T1R3: (**a**) homology model of the T1R2/T1R3 heterodimer; (**b**) Ramachandran plot of the T1R2/T1R3 heterodimer homology model; (**c**) homology models of the individual T1R2 and T1R3 receptor subunits; (**d**) Ramachandran plots of the T1R2 and T1R3 homology models. In the Ramachandran plots, the letters are standard region labels for different backbone dihedral-angle conformational regions; uppercase and lowercase letters indicate favored and allowed regions, respectively. T1R2, taste receptor type 1 member 2; T1R3, taste receptor type 1 member 3.

Given that the licorice extract exhibited pronounced lingering sweetness characteristics during sensory evaluation, homology models of the human sweet taste receptors T1R2 and T1R3 were developed. These models aim to facilitate a molecular-level exploration of the taste mechanism of GA, the core component, as well as the intervention mechanisms of polyphenols. This structural foundation allows for the subsequent elucidation of interaction modes between flavour molecules and receptor proteins. The specific results and evaluations of the modeling process are as follows:

Sweet taste receptors belong to the class of GPCRs [49]. The receptor is composed of two subunits, T1R2 and T1R3. The 3D structure of the T1R2/T1R3 heterodimer, obtained through homology modeling, is illustrated in Figure 6a. The model was evaluated using Verify 3D and Ramachandran plots. The Verify3D score was 41.93%. As presented in the Ramachandran plot in Figure 6b, 89.25% of the amino acid residues were situated in favored regions, while 2.78% were in allowed regions, and only 1.67% were in outlier regions. These findings are consistent with results from previous studies, demonstrating that the established model is reasonable and suitable for investigating the sweetness mechanism and intervention strategies of GA [32,43].

To further investigate the relationships between GA and individual subunits, homology modeling of the T1R2 and T1R3 subunits was conducted using Modeller 10.4. The resulting structures are presented in Figure 6c, where both receptor proteins displayed the typical structural characteristics of GPCRs. The SAVES v6.0 server was utilized to evaluate the two obtained protein models; the Ramachandran plots of these models are presented in Figure 6d. The Verify3D scores for the T1R2 and T1R3 models were 36.47% and 63.73%, respectively. Specifically, 88.3% and 90.3% of the amino acid residues were located in the favored regions, 10.8% and 8.3% in the allowed regions, and 0.9% and 0.7% in the outlier regions for the T1R2 and T1R3 models, respectively. The evaluation results indicated that more than 98% of the residues in both protein models fell within the favored and allowed regions, demonstrating that the models are reasonable and suitable for subsequent molecular docking studies.

### 3.8. Molecular Mechanism of Sweetness of GA

**Figure 7:**
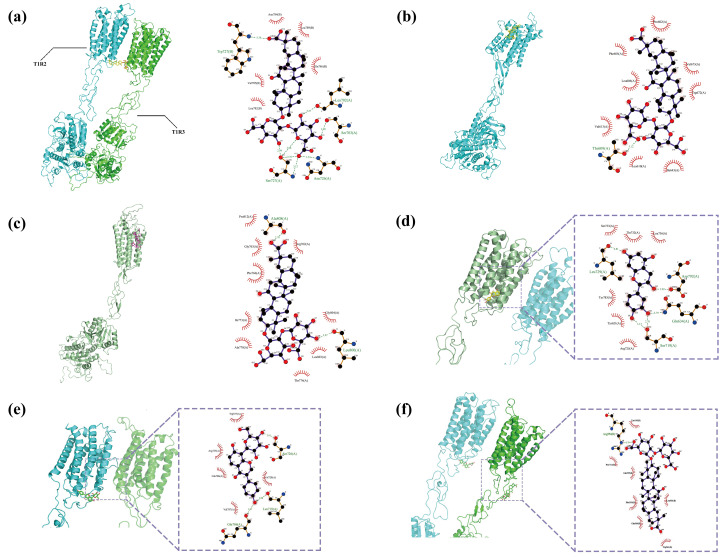
Molecular docking of ligands and the competitive mechanism of puerarin: (**a**) interaction sites of GA with the sweet taste receptor model; (**b**,**c**) binding sites of GA with T1R2 and T1R3 subunit models; (**d**) binding interaction of catechin with the sweet taste receptor; (**e**) binding interaction of puerarin with the sweet taste receptor; (**f**) binding of GA with the sweet taste receptor-puerarin complex. GA, glycyrrhizic acid; T1R2, taste receptor type 1 member 2; T1R3, taste receptor type 1 member 3.

Previous reports have suggested that the binding of most sweet molecules occurs at the Venus Flytrap (VFT) domains of T1R2 and T1R3. In this study, GA was docked with the receptor protein models, and the results are illustrated in Figure 7a. It was observed that GA bound to the extracellular region of the TMD, specifically within the cavities formed by the amino acid helical structures of the receptor dimer. In a previous study, Schmid et al. docked various saponins from licorice with the VFT domain of the T1R2 receptor, where GA yielded a docking score of −8.93 [5,50]. The absolute value of this score was lower than the score of −9.80 obtained for the TMD region in the current study, indicating that GA molecules possess a higher affinity for binding with the TMD region. Prior research has indicated that the interaction between GA and sweet taste receptors is primarily driven by hydrogen bonds and hydrophobic forces, with a reported binding energy of −41.00 kJ/mol [51,52]. In the present study, the interaction between GA and the T1R2 receptor was primarily mediated by hydrogen bonds formed at residues Ser727, Asn726, Ser703, and Leu702. The lengths of these hydrogen bonds ranged from 2.76 to 3.27 Å. In contrast, GA interacted with the T1R3 receptor predominantly through hydrophobic forces involving residues Leu782, Val785, Asn784, Leu789, and Gln786. Additionally, a hydrogen bond was formed at position Trp727 with a bond length of 3.36 Å. In conclusion, the binding affinity of GA for the T1R3 protein was stronger than that for T1R2 [53]. This T1R3 subunit preference mirrors the binding patterns of other high-potency sweeteners, such as cyclamate and neohesperidin dihydrochalcone (NHDC), which also interact primarily with the TMD of T1R3. While smaller intense sweeteners often involve more transient VFT interactions, the deep burial of GA within the transmembrane helical bundle explains its distinctive kinetic persistence compared to these traditional sweeteners [14].

Various explanations currently exist for the lingering sweetness effect of sweeteners. Some researchers argue that sweetness intensity and duration are directly correlated with the binding affinity between the sweetener molecule and its receptor. For instance, a more negative binding free energy (ΔG) is associated with higher sweetness intensity [49]. When the ΔG is extremely low, indicating high stability, the dissociation rate of the molecule from the taste receptors slows down. This leads to the continuous transmission of sweetness signals, which manifests as lingering sweetness perception [54]. Singla et al. suggested that the dimensions of the binding pocket influence the entry and exit of sweetener molecules, thereby modulating taste response times and durations [55]. Given that the binding sites for GA are situated within the cavities of the helical structures, this mechanism appears analogous to that of bitterness receptors (T2Rs), where the influence of helical domains on perception duration is most prominent. In T2Rs, bitterness molecules must penetrate the interior of the receptor helix to elicit a response, resulting in a slow onset and prolonged duration [55,56]. Similarly, the restricted space available for GA to enter its binding site results in a slower onset of sweetness perception. Meanwhile, the significant steric hindrance encountered during the dissociation of the molecule contributes to the prolonged sweetness duration of GA. Furthermore, for a given sweet substance, both sweetness duration and intensity are positively correlated with its concentration [54]. Consequently, it remains difficult to definitively determine whether the lingering sweetness of GA is a sensory retardation caused by excessive receptor stimulation from high intensity, or a result of slow dissociation due to robust intermolecular interactions.

To further validate the interactions between GA and the two receptor subunits, the T1R2 and T1R3 subunits were docked with GA individually. The docking results are illustrated in Figure 7b,c. The binding energy between T1R2 and GA was −25.104 kJ/mol, involving the formation of a hydrogen bond at residue Thr609 with a bond length of 3.12 Å. Additionally, hydrophobic interactions were observed at eight residues, specifically Pro602, Phe605, Leu606, Val613, Leu610, Ile643, Trp672, and Val673. For T1R3, the binding energy with GA was −27.196 kJ/mol. Two hydrogen bonds were formed at residues Ala808 and Leu800, with bond lengths of 2.70 Å and 3.07 Å, respectively. Hydrophobic interactions occurred at nine residues, including Pro812 and Gly763. The binding affinity for T1R3 was more favorable than that for T1R2, which was consistent with the previously obtained docking results for the heterodimer. Sweetness duration is not determined by receptor binding affinity alone, but is also influenced by matrix-related, physiological, and perceptual factors. Nevertheless, the predicted receptor-binding features of GA identified in this study may help explain its prolonged sweetness duration at the receptor level. This interpretation is consistent with previous studies linking sweetener structure and receptor binding sites to temporal sweetness profiles [13,54]. Together, the more favorable predicted binding energy of GA with T1R3 and its predicted location within the TMD region suggest a relatively stable GA–receptor binding state, which may contribute to the longer sweetness duration of GA compared with sucrose.

### 3.9. Intervention Mechanisms of Polyphenols on the Sweetness of GA

According to the findings in Section 3.6, puerarin and catechin exhibited superior effects in mitigating the lingering sweetness perception of the licorice extract. Consequently, molecular docking was performed between these two polyphenols and the TMD region of the receptor protein models to explore the intervention mechanisms underlying the lingering sweetness [57]. The docking results are presented in Figure 7d,e. The catechin molecule was found to interact with the TMD region of the T1R3 receptor with a binding energy of −31.80 kJ/mol, forming five hydrogen bonds and six points of interaction. The absolute value of this binding energy was lower than that of the interaction between GA and the receptor, suggesting that catechin does not substantially interfere with the binding of GA to the sweet taste receptor protein, or its impact is minimal. Interestingly, research has indicated that typical bitter and astringent substances in tea, such as catechin and EGCG, possess “Hui gan” (lingering sweet aftertaste) properties, where the intensity of the sweetness is proportional to the addition level [15,44]. This suggests that catechin has the potential to bind with sweet taste receptors and elicit a sweetness perception. Humans perceive the bitterness of catechin through the bitter taste receptors TAS2R4, TAS2R5, and TAS2R39 [58]. These receptors feature a helical domain composed of seven transmembrane helices, which is structurally similar to the TMD region in the present model. This similarity further supports the potential for catechin to bind with the TMD region of sweet taste receptors.

The docking results of puerarin with the TMD region of the sweet taste receptor indicated its capability to bind with the T1R2 receptor. The binding energy was −52.72 kJ/mol, which was more favorable than the binding energy between GA and the sweet taste receptor (−41.00 kJ/mol). Consequently, puerarin may act upon the receptor protein, thereby modulating the sweetness duration of GA. Although no studies have reported the binding of puerarin to sweet taste receptors to date, pharmacological research has demonstrated that the target receptors of puerarin include various GPCRs, with binding sites situated at the upper end of the helical domain [59,60]. These findings are broadly consistent with the docking results obtained in the present study.

The taste receptor-puerarin complex was docked with GA to explore the possible influence of puerarin on GA binding to the sweet taste receptor [57]. The results are illustrated in Figure 7f. The presence of puerarin led to the occupation of the original binding site of GA, which was previously situated within the interior of the T1R2 TMD helical structure. Accordingly, the predicted binding pose of GA shifted from the TMD interior to the outer region of the T1R3 TMD. A hydrogen bond with a length of 2.79 Å was formed at residue Arg554. Concurrently, seven hydrophobic interaction sites were identified at residues Tyr545, Pro713, Gln555, Phe558, Glu560, Trp561, and Leu556. This site shift was accompanied by a change in the docking energy of GA from −41.00 kJ/mol to −21.30 kJ/mol, suggesting a weaker predicted interaction between GA and the receptor in the presence of puerarin. This change is consistent with the sensory observation that puerarin shortened the sweetness duration of licorice extract. Together with the stronger predicted interaction of puerarin with the TMD region compared with catechin, these results support a plausible receptor-level explanation for the stronger effect of puerarin in reducing GA-related lingering sweetness. Therefore, puerarin may be considered a promising candidate for mitigating the lingering sweetness of licorice extract in formulated spirits.

## 4. Conclusions

This study evaluated the sensory characteristics of licorice extract in spirits with different ethanol concentrations and explored a possible receptor-level mechanism underlying the lingering sweetness of GA. The suitable addition levels of licorice extract were 30 mg/L in 42% vol spirits and 40 mg/L in 52% vol spirits, while the upper comfort limits were 75.13 mg/L and 72.98 mg/L, respectively. These values define a practical concentration range for blending and may help avoid excessive lingering sweetness or sensory discomfort. In addition, the GA-equivalent relative sweetness was 175.83 times that of sucrose, indicating the potential of licorice extract as a high-potency sweetening ingredient in spirit formulations with reduced sugar content.

Although puerarin showed a clear effect in mitigating the characteristic sweetness persistence of GA, several limitations should be acknowledged. First, this study used one licorice extract containing 23.75% GA and tested only three selected polyphenols. The sensory outcomes may therefore vary with extract composition and the type of sweetness modulator. Although panel consistency was validated, age, sex, drinking habits, and sweetness sensitivity were not analyzed separately, despite their potential influence on sweetness perception. Future studies should standardize GA content, include more candidate modulators, and use larger stratified panels for validation in target beverage matrices. Second, molecular docking provided a plausible receptor-level explanation for the effect of puerarin, but it remains a static prediction approach. Further validation using molecular dynamics simulations, receptor assays, and binding kinetics studies is still needed. Third, the industrial application of puerarin in commercial formulated spirits requires further evaluation of regulatory compliance, sensory compatibility, storage stability, cost-effectiveness, and long-term consumer acceptance before practical scale-up.

Taken together, this study links molecular interactions with sensory performance and provides an integrated framework for understanding and modulating the lingering sweetness of GA in systems containing ethanol. The findings may guide the rational use of licorice extract and other botanical functional ingredients in alcoholic beverages, thereby supporting the development of formulated spirits with improved sensory consistency, quality stability, and reduced sugar content across different ethanol matrices.

## Data Availability

The original contributions presented in this study are included in the article. Further inquiries can be directed to the corresponding author.

## References

[1] Yang L., Jiang Y., Zhang Z., Hou J., Tian S., Liu Y. (2020). The Anti-Diabetic Activity of Licorice, a Widely Used Chinese Herb. J. Ethnopharmacol..

[2] Yang R., Yuan B.-C., Ma Y.-S., Zhou S., Liu Y. (2017). The Anti-Inflammatory Activity of Licorice, a Widely Used Chinese Herb. Pharm. Biol..

[3] Wang H., Song W., Tao W., Zhang J., Zhang X., Zhao J., Yong J., Gao X., Guo L. (2021). Identification Wild and Cultivated Licorice by Multidimensional Analysis. Food Chem..

[4] Sabbioni C., Mandrioli R., Ferranti A., Bugamelli F., Saracino M., Forti G., Fanali S., Raggi M. (2005). Separation and Analysis of Glycyrrhizin, 18β-Glycyrrhetic Acid and 18α-Glycyrrhetic Acid in Liquorice Roots by Means of Capillary Zone Electrophoresis. J. Chromatogr. A.

[5] Schmid C., Brockhoff A., Ben Shoshan-Galeczki Y., Kranz M., Stark T.D., Erkaya R., Meyerhof W., Niv M.Y., Dawid C., Hofmann T. (2021). Comprehensive Structure-Activity-Relationship Studies of Sensory Active Compounds in Licorice (*Glycyrrhiza glabra*). Food Chem..

[6] Graebin C.S., Merillon J., Ramawat K. (2018). The Pharmacological Activities of Glycyrrhizinic Acid (“glycyrrhizin”) and Glycyrrhetinic Acid. Sweeteners: Pharmacology, Biotechnology, and Applications.

[7] (2024). National Food Safety Standard—Standard for Uses of Food Additives.

[8] Wan J., Zhang Y., Huang P., Fan H., Ding Z. (2017). Study on Crude Extraction Technology of Glycyrrhizic Acid in Licorice. Chin. Arch. Tradit. Chin. Med..

[9] Saraiva A., Carrascosa C., Raheem D., Ramos F., Raposo A. (2020). Natural Sweeteners: The Relevance of Food Naturalness for Consumers, Food Security Aspects, Sustainability and Health Impacts. Int. J. Environ. Res. Public Health.

[10] (2021). Terminology and Classification of Beverages and Alcoholic Beverages.

[11] Keast S., Breslin P. (2003). An Overview of Binary Taste-Taste Interactions. Food Qual. Prefer..

[12] Sharifi-Rad J., Quispe C., Herrera-Bravo J., Belen L.H., Kaur R., Kregiel D., Uprety Y., Beyatli A., Yeskaliyeva B., Kirkm C. (2021). Glycyrrhiza Genus: Enlightening Phytochemical Components for Pharmacological and Health-Promoting Abilities. Oxid. Med. Cell. Longev..

[13] Karl C.M., Wendelin M., Lutsch D., Schleining G., Duerrschmid K., Ley J.P., Krammer G.E., Lieder B. (2020). Structure-Dependent Effects of Sweet and Sweet Taste Affecting Compounds on Their Sensorial Properties. Food Chem. X.

[14] Hao S., Guthrie B., Kim S.-K., Balanda S., Kubicek J., Murtaza B., Khan N.A., Khakbaz P., Su J., Goddard W.A. (2024). Steviol Rebaudiosides Bind to Four Different Sites of the Human Sweet Taste Receptor (T1R2/T1R3) Complex Explaining Confusing Experiments. Commun. Chem..

[15] Zhang Y.-N., Yin J.-F., Chen J.-X., Wang F., Du Q.-Z., Jiang Y.-W., Xu Y.-Q. (2016). Improving the Sweet Aftertaste of Green Tea Infusion with Tannase. Food Chem..

[16] (2023). Quality Requirements for Edible Alcohol.

[17] Wu S., Gong G., Wang Y., Li F., Jia S., Qin F., Ren H., Liu Y. (2013). Response Surface Optimization of Enzyme-Assisted Extraction Polysaccharides from Dictyophora Indusiata. Int. J. Biol. Macromol..

[18] Sun P., Liu X., Pan Q., Zhang X., He S. (2020). Comparison of Chinese Licorice (*Glycyrrhiza uralensis*) Granules and Water Extracts and Investigation of Their Antibacterial Activities for Veterinary Application. Eur. J. Integr. Med..

[19] Zhang L., Huang Z., Luo S., Cao L., Xie Y., Qian J. (2023). Establishment of Non-Targeted Screening Database and Confirmation Method for 18 Mycotoxins in Grains Using Ultra Performance Liquid Chromatography Quadrupole-Time of Flight Mass Spectrometry. Chin. J. Chromatogr..

[20] (2012). Sensory Analysis—General Guidelines for the Selection, Training and Monitoring of Selected Assessors—Part 1: Selected Assessors.

[21] (2022). Sensory Analysis—Methodology—Initiation and Training of Assessors in the Detection and Recognition of Odours.

[22] (2012). Sensory Analysis—Methodology—Methods for Determination of Sensitivity of Taste.

[23] (2012). Sensory Analysis—Methodology—General Guidance.

[24] (2016). Terminology of Sensory Evaluation of Baijiu.

[25] (2016). Guidelines for Sensory Evaluation of Baijiu.

[26] Zhang C., He F., Li Z., He J. (2022). Classification of Base Liquor of Sauce-Flavor Baijiu Based on Principal Component Analysis and Hierarchical Clustering Analysis. China Brew..

[27] Kokkinidou S., Peterson D.G. (2016). Identification of Compounds That Contribute to Trigeminal Burn in Aqueous Ethanol Solutions. Food Chem..

[28] Kim M.-J., Yoo S.-H., Kim Y., Hong J.-H. (2016). Relative Sweetness and Sweetness Quality of Phyllodulcin [(3R)-8-Hydroxy-3-(3-Hydroxy-4-Methoxyphenyl)-3,4-Dihydro-1H-Isochrome n-1-One]. Food Sci. Biotechnol..

[29] Jung J., Kim S., Park S., Hong J.-H. (2021). Sweetness Profiles of Glycosylated Rebaudioside A and Its Binary Mixtures with Allulose and Maltitol. Food Sci. Biotechnol..

[30] Yang Q., Ng M.L., Rogers L. (2017). Chapter 5—Paired Comparison/Directional Difference Test/2-Alternative Forced Choice (2-AFC) Test, Simple Difference Test/Same-Different Test. Discrimination Testing in Sensory Science;Woodhead Publishing Series in Food Science, Technology and Nutrition.

[31] Nagai M., Matsumoto S., Endo J., Sakamoto R., Wada M. (2015). Sweet Taste Threshold for Sucrose Inversely Correlates with Depression Symptoms in Female College Students in the Luteal Phase. Physiol. Behav..

[32] Ebbeling C.B., Feldman H.A., Steltz S.K., Quinn N.L., Robinson L.M., Ludwig D.S. (2020). Effects of Sugar-sweetened, Artificially Sweetened, and Unsweetened Beverages on Cardiometabolic Risk Factors, Body Composition, and Sweet Taste Preference: A Randomized Controlled Trial. J. Am. Heart Assoc..

[33] Miele N.A., Cabisidan E.K., Plaza A.G., Masi P., Cavella S., Di Monaco R. (2017). Carbohydrate Sweetener Reduction in Beverages through the Use of High Potency Sweeteners: Trends and New Perspectives from a Sensory Point of View. Trends Food Sci. Technol..

[34] Yaroshenko I., Kirsanov D., Kartsova L., Sidorova A., Sun Q., Wan H., He Y., Wang P., Legin A. (2016). Exploring Bitterness of Traditional Chinese Medicine Samples by Potentiometric Electronic Tongue and by Capillary Electrophoresis and Liquid Chromatography Coupled to UV Detection. Talanta.

[35] Li X., Staszewski L., Xu H., Durick K., Zoller M., Adler E. (2002). Human Receptors for Sweet and Umami Taste. Proc. Natl. Acad. Sci. USA.

[36] Sun Y., Zhang S., Bao T., Jiang Z., Huang W., Xu X., Qiu Y., Lei P., Wang R., Xu H. (2025). Comprehensive New Insights into Sweet Taste Transmission Mechanisms and Detection Methods. Foods.

[37] Zhao S., Zheng H., Lu Y., Zhang N., Soladoye O.P., Zhang Y., Fu Y. (2023). Sweet Taste Receptors and Associated Sweet Peptides: Insights into Structure and Function. J. Agric. Food Chem..

[38] Brasser S.M., Norman M.B., Lemon C.H. (2010). T1r3 Taste Receptor Involvement in Gustatory Neural Responses to Ethanol and Oral Ethanol Preference. Physiol. Genom..

[39] Thibodeau M., Bajec M., Pickering G. (2017). Orosensory Responsiveness and Alcohol Behaviour. Physiol. Behav..

[40] Cong X., Wu Z., Wu J., Huang M., Sun W., Sun Y., Zhao D., Zheng F. (2026). Ethanol Regulates Bitterness Perception of the Trp-Ile-Lys-Lys (WIKK) Peptide by Activating the Human Bitter Receptor T2R47. Foods.

[41] Burke M.V., Small D.M. (2015). Physiological Mechanisms by Which Non-Nutritive Sweeteners May Impact Body Weight and Metabolism. Physiol. Behav..

[42] Reyes M.M., Castura J.C., Hayes J.E. (2017). Characterizing Dynamic Sensory Properties of Nutritive and Nonnutritive Sweeteners with Temporal Check-All-That-Apply. J. Sens. Stud..

[43] Wang L., Luo Z., Liu D. (2026). Dietary Antioxidants, Polyphenols, and Vascular Health: Insights From Ultrasound Measurement of Carotid Intima-Media Thickness and Their Association With Cognitive Function in Aging and Neurodegenerative Diseases. Phytother. Res..

[44] Chong P.H., Chen J., Yin D., Qin L. (2022). Tea Compound-Saliva Interactions and Their Correlations with Sweet Aftertaste. npj Sci. Food.

[45] Xue G., Zhou T., Li M., Yu Q., Deng Z., Lin J., Zheng C., Han L., Zhang D., Huang H. (2025). Sweet Aftertaste (Huigan) in Foods: Evaluation and Processing Impacts for Enhanced Flavour Design and Applications. Compr. Rev. Food Sci. Food Saf..

[46] Wang Z., Yang B., Zhou P., Yang G., Zhao Z. (2025). Study on the Effects and Mechanisms of Action of Biological Enzymes on the Quality of Summer Rock Tea Extract. Appl. Sci..

[47] Maaroufi H. (2024). Novel Gurmarin-like Peptides from Gymnema Sylvestre and Their Interactions with the Sweet Taste Receptor T1R2/T1R3. Chem. Senses.

[48] Ickes C.M., Cadwallader K.R. (2017). Effects of Ethanol on Flavor Perception in Alcoholic Beverages. Chemosens. Percept..

[49] Masuda K., Koizumi A., Nakajima K., Tanaka T., Abe K., Misaka T., Ishiguro M. (2012). Characterization of the Modes of Binding between Human Sweet Taste Receptor and Low-Molecular-Weight Sweet Compounds. PLoS ONE.

[50] Hsin K.-Y., Ghosh S., Kitano H. (2013). Combining Machine Learning Systems and Multiple Docking Simulation Packages to Improve Docking Prediction Reliability for Network Pharmacology. PLoS ONE.

[51] Jiang P., Cui M., Zhao B., Snyder L., Benard L., Max M., Margolskee R. (2005). Identification of the Cyclamate Interaction Site within the Transmembrane Domain of the Human Sweet Taste Receptor Subunit T1R3. J. Biol. Chem..

[52] Mayank, Jaitak V. (2015). Interaction Model of Steviol Glycosides from *Stevia rebaudiana* (Bertoni) with Sweet Taste Receptors: A Computational Approach. Phytochemistry.

[53] Morita A., Omoya Y., Ito R., Ishibashi Y., Hiramoto K., Ohnishi S., Yoshikawa N., Kawanishi S. (2021). Glycyrrhizin and Its Derivatives Promote Hepatic Differentiation via Sweet Receptor, Wnt, and Notch Signaling. Biochem. Biophys. Rep..

[54] Deck C.M., Behrens M., Wendelin M., Ley J.P., Krammer G.E., Lieder B. (2022). Impact of Lactisole on the Time-Intensity Profile of Selected Sweeteners in Dependence of the Binding Site. Food Chem. X.

[55] Singla R., Jaitak V. (2016). Synthesis of Rebaudioside A from Stevioside and Their Interaction Model with hTAS2R4 Bitter Taste Receptor. Phytochemistry.

[56] Xu W., Wu L., Liu S., Liu X., Cao X., Zhou C., Zhang J., Fu Y., Guo Y., Wu Y. (2022). Structural Basis for Strychnine Activation of Human Bitter Taste Receptor TAS2R46. Science.

[57] Li H., Li C. (2010). Multiple Ligand Simultaneous Docking: Orchestrated Dancing of Ligands in Binding Sites of Protein. J. Comput. Chem..

[58] Soares S., Kohl S., Thalmann S., Mateus N., Meyerhof W., De Freitas V. (2013). Different Phenolic Compounds Activate Distinct Human Bitter Taste Receptors. J. Agric. Food Chem..

[59] Cheng Y.-F., Zhu G., Wu Q.-W., Jiang Y., Guo L., Guan Y.-L., Liu Y.-S., Zhang J. (2017). GPR30 Activation Contributes to the Puerarin-Mediated Neuroprotection in MPP^+^-Induced SH-SY5Y Cell Death. J. Mol. Neurosci..

[60] Pham T.H., Lee G.H., Jin S.W., Lee S.Y., Han E.H., Kim N.D., Jeong H.G. (2022). Puerarin Attenuates Hepatic Steatosis via G-Protein-Coupled Estrogen Receptor-Mediated Calcium and SIRT1 Signaling Pathways. Phytother. Res..

